# Another Case

**Published:** 1860-01

**Authors:** 


### ANOTHER CASE.

BY THE SENIOR EDITOR.

In connection with the preceding, we give the following case as not devoid of interest:

Mrs. K., aged about 20 years, short in stature, full muscular development, and sanguine temperament; had passed through the full period of her first pregnancy in good health as her friends supposed, until the morning of the 7th of November, 1859. On that morning, it was observed that her face and neck were bloated more than usual; she had had frequent desire to urinate during the preceeding night; and some pain in the occipital region for several days. Between 12 and 1 o'clock of that day, she was suddenly seized with violent convulsions. I was hastily summoned to her bed-side, and found her just recovering from the state of insensibility which had followed the first paroxysm of convulsions. On inquiry, we learned that she had been troubled with disuria, and more or less edematous swelling of the face, neck, and extremities, for two or three weeks. She had no appearance of uterine pains, or vaginal discharge. The mouth of the uterus would scarcely admit the end of the finger, and the os was not soft or yielding to pressure. The temperature of the surface being increased, the head giddy and somewhat painful, and the pulse full and firm, I thought it necessary to abstract blood. While waiting for a bandage and bowl, another convulsion occurred very suddenly, and violently agitated the whole muscular system, until the face became very purple, and the pulse small and frequent. As soon as the muscular agitation had abated sufficiently, I opened a vain in the arm, and allowed the blood to flow until more than thirty ounces had been abstracted. The pulse becoming soft, the face pale, and the breathing more natural, the arm was tied up, and a powder, of calomel 10 grs. with 10 grs. of nitrate of potassa, was immediately administered.

The patient had vomited freely after the first paroxysm, and the vomiting recurred slightly soon after swallowing the powder. In half an hour after the bleeding, the patient recovered her consciousness, said her head felt better, and the respiration and circulation became so much improved that we began to hope no more paroxysms would recur.

In less than an hour, however, another paroxysm of convulsion came on so suddenly that, though we were watching for that purpose, we were unable to anticipate it sufficiently to bring the patient under the influence of chloroform; and it proved quite as severe as either of the preceding ones. Feeling now confident that the paroxysms would recur until the patient was delivered, and the os uteri having become more soft and yielding, at my request Prof. Byford was called to advise in reference to the propriety of rupturing the membranes, with a view of inducing efficient pains and hastening delivery. Soon after his arrival, a fourth paroxysm occurred, during which the bandage was displaced from the arm, and another pint of blood was lost. She now remained unconscious, the pulse 120 per minute and small, the breathing noisy, and the deglutition difficult. Prof. Byford ruptured the membranes, and a considerable quantity of liquor amnii escaped, followed by regular, but not forcible, uterine contractions. At his suggestion, one drop of croton oil was administered every hour to induce speedy and free alvine evacuations, and the patient closely watched, in order to prevent the further convulsions by the inhalation of chloroform. Notwithstanding these precautions, however, the convulsive paroxysms continued to recur as often as every hour and a half; and as the os uteri became entirely relaxed, while the uterine contractions were inefficient, we gave a drachm of the fluid extract of ergot every half hour. Enemas were also used to hasten a movement of the bowels. Still, the labor advanced so slowly, that at ten o'clock in the evening, the head had but just fairly engaged in the superior straight of the pelvis, and the os frontis was found to be towards the pubes. In the meantime the pulse had increased to 150 per minute, was small and soft; the breathing somewhat stertorous, and the patient wholly unconscious. With the sanction of Prof. Byford, we now determined to complete delivery without further delay; and though the head was still high in the pelvis, we succeeded in adjusting Davis's long forceps to the head of the child, and at half-past 10 o'clock, P. M., she was delivered of a full sized male child, but entirely lifeless ; there being no pulsation either in the cord or the heart. The placenta was soon after expelled; the uterus contracted down firmly, and the hemorrhage was very slight. The patient being now very restless and feeble, we gave one quarter of a grain of sulphate of morphine, with the intention of repeating it every two hours. During the two succeeding hours she became gradually more quiet, the breathing more natural, the pulse a little less frequent, and we began to flatter ourselves that the danger was past, when suddenly another paroxysm of convulsions ensued, more violent, if possible, than those that had preceded delivery. They now continued to recur at short intervals until 4½ o'clock, A. M., in spite of the most assiduous use of chloroform, aided by anodyne enemas and sponging of the whole surface of the body with warm vinegar.

At half-past four o'clock in the morning of the 8th of Nov., the spasmodic action ceased, but the patient was left in a state of extreme exhaustion. The pulse was too small and frequent to be counted; the skin cool and covered with a clammy sweat; her countenance bloated and livid; and her breathing very stertorous and noisy from the abundance of mucous in the trachea and bronchial tubes, considerable quantities of which were forced out in a frothy condition, both from the mouth and nostrils. Deglutition was entirely suspended, and for half an hour the lower jaw was dropped and involuntarily thrust forward with each prolonged expiration like one dying. From this condition she recovered very gradually, being only able to swallow with difficulty at 6 o'clock, A. M. Apprehending serous effusion into the cavities of the brain, and infiltration of the lungs, from the severity and duration of the preceding spasms, we directed the following:

℞ Fluid Extract of Scuttellaria, ℥j.
Iodide of Potassa, ʒjj.

Take twenty drops every hour. Bottles of hot water were applied to the extremities, and as there had been no movement of the bowels, two more drops of croton oil were given during the morning. Her respiration and circulation improved very slowly during all the forenoon. Still she remained wholly unconscious, respiration noisy, and deglutition difficult, until 3 o'clock, P. M., when the pulse again became extremely rapid and feeble, with symptoms of immediate fatal prostration. Having some whiskey in the house, we hastily mixed equal parts of milk, water and whiskey with some sugar, and succeeded in getting the patient to swallow three teaspoonfuls of it every fifteen minutes.

Soon after the third day she began to improve. The respiration was less noisy, and the pulse slower. Prof. Byford was again called in, and on his suggestion, gin in place of whisky was substituted, and the punch in doses of two dessert spoonfuls, was continued every half hour, and sponging of the surface with warm vinegar was repeated. No urine having passed since the patient was sick, we introduced the catheter about 11 o'clock, A. M., and drew off a pint of pretty clear urine.

The gin punch and sponging with vinegar was continued until the morning of the 9th, when the respiration and deglutition had become quite easy, and the pulse was reduced to 120 per minute. The bowels moved for the first time during the night, though she had taken six or seven drops of croton oil nearly twenty-four hours previously. Still the patient remained entirely unconscious. I again introduced the catheter, and removed nearly a quart of high colored urine. The punch was continued through the day once an hour, and one or two table spoonsful of chicken broth, between each of the doses. During the day the bowels moved so freely that two or three moderate doses of morphine were given to quiet them. In the evening, she for the first time since the afternoon of the 7th, opened her eyes and looked around with a wild or bewildered expression of countenance. During the night she voided the urine twice, and rested quietly much of the time. On the 10th, three full days from the attack, she for the first time became sufficiently conscious to recognize her friends. The pulse having regained a fair degree of strength, and the urinary secretion being free, the stimulant was gradually withdrawn, and the amount of simple nourishment increased. The lochial discharge was scanty but natural, and from this time the patient rapidly recovered.

